# Supplementary material for: A method for explaining individual predictions in neural networks
Source: PeerJ Comput Sci. 2025 Apr 7;11:e2802. doi: 10.7717/peerj-cs.2802 (PMC12190433; doi:10.7717/peerj-cs.2802)

Algorithm 1 (NNexplainer) Example

BASE neural network

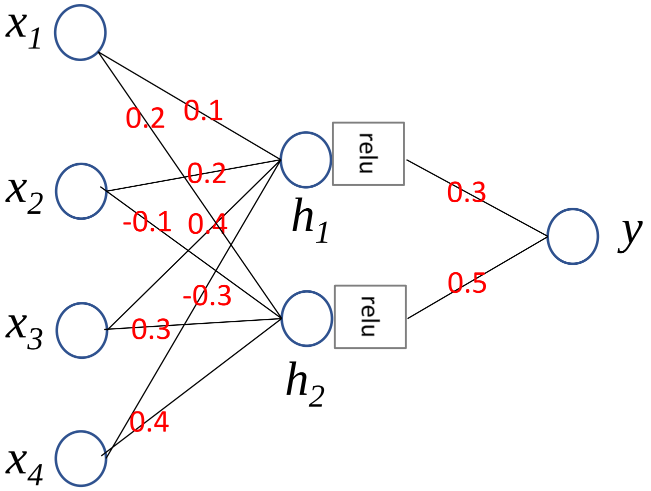

$$\mathbf{W_o} = \begin{pmatrix} 0.3 \\ 0.5 \end{pmatrix}$$

$$\mathbf{W_I} = \begin{pmatrix} 0.1 & 0.2 \\ 0.2 & -0.1 \\ 0.4 & 0.3 \\ -0.3 & 0.4 \end{pmatrix}$$

Task

Show the contribution of x_1, x_2, x_3, and x_4 to the output y when the input values $\boldsymbol{x}$ are as follows:

$$\text{Input } \boldsymbol{x} = \begin{bmatrix} 0.6 & 0.5 & 0.5 & 0.1 \end{bmatrix}$$

Algorithm

Algorithm 1. Calculation of contribution of input values

```
Inputs:
   test_input       // input data for prediction
   weights          // list of weight matrixes of predictive model
   N                // number of layers
Output:
   cont_list        // list of contribution values for each feature

BEGIN

   // build a list of transformation rate for each layer
   TR = []                              // transformation rate
   FOR ln FROM 0 To (N-1)
      calculate weighted sum WS of layer ln
       calculate layer_outputs of layer ln       // layer_outputs = ϕ(WS)
       TR_tmp := layer outputs / WS
      append TR_tmp to TR
   END FOR

   // calculate contribution of input values
   cont_matrix_before := diag(test_input)   // diagonal of test_input

   FOR ln FROM 0 to (N-1)
      // Calculate Cx for given layer
      cont_matrix_this := mat_mul(WEIGHTS[ln].T, cont_matrix_before)
      cont_matrix_before  := cont_matrix_this  ⊗ TR[ln]
   END FOR

   cont_list = cont_matrix_before
   RETURN(cont_list)

END
```

```
Inputs:
   test_input      // input data for prediction
   weights         // list of weight matrixes of predictive model
   N               // number of layers
Output:
   cont_list      // list of contribution values for each feature
```

```
test_input = [0.6, 0.5, 0.5, 0.1]
weights    = [ [ 0.1, 0.2
                 0.2,-0.1
                 0.4, 0.3
                -0.3, 0.4 ] ,
               [ 0.3
                 0.5 ]
             ]
N = 2
```

```
// build a list of transformation rate for each layer
TR = []                              // transformation rate
FOR ln FROM 0 To (N-1)
   calculate weighted sum WS of layer ln
    calculate layer_outputs of layer ln       // layer_outputs = ϕ(WS)
    TR_tmp := layer outputs / WS
   append TR_tmp to TR
END FOR
```

ln = 0

$$WS = [0.6,\ 0.5,\ 0.5,\ 0.1] \bullet \begin{pmatrix} 0.1, & 0.2 \\ 0.2, & -0.1 \\ 0.4, & 0.3 \\ -0.3, & 0.4 \end{pmatrix} = [0.33,\ 0.26]$$

$$layer\_outputs = \mathit{relu}([0.33,\ 0.26]) = [0.33,\ 0.26]$$

$$TR\_tmp = [0.33,\ 0.26] \ / \ [0.33,\ 0.26] = [1,\ 1]$$

$$TR = [[1,1]]$$

---

ln = 1

$$WS = [0.33,\ 0.26] \bullet \begin{pmatrix} 0.3 \\ 0.5 \end{pmatrix} = [0.229]$$

$$layer\_outputs = \mathit{relu}([0.229]) = [0.229]$$

$$TR\_tmp = [0.229] \ / \ [229] = [1]$$

$$TR = [[1,1],[1]]$$

```
// calculate contribution of input values
cont_matrix_before := diag(test_input)   // diagonal of test_input
```

```
test_input = [0.6, 0.5, 0.5, 0.1]
```

$$
\text{cont\_matrix\_before} = \begin{pmatrix} 0.6, & 0, & 0, & 0 \\ 0, & 0.5, & 0, & 0 \\ 0, & 0, & 0.5, & 0 \\ 0, & 0, & 0, & 0.1 \end{pmatrix}
$$

```
FOR ln FROM 0 to (N-1)
   // Calculate Cx for given layer
   cont_matrix_this := mat_mul(WEIGHTS[ln].T, cont_matrix_before)
   cont_matrix_before := cont_matrix_this ⊗ TR[ln]
END FOR
```

ln = 0

$$\text{cont\_matrix\_this} = \begin{bmatrix} 0.1, & 0.2, & 0.4, & -0.3 \\ 0.2, & -0.1, & 0.3, & 0.4 \end{bmatrix} \bullet \begin{bmatrix} 0.6, & 0, & 0, & 0 \\ 0, & 0.5, & 0, & 0 \\ 0, & 0, & 0.5, & 0 \\ 0, & 0, & 0, & 0.1 \end{bmatrix} = \begin{bmatrix} 0.06, & 0.1, & 0.2, & -0.03 \\ 0.12, & -0.05, & 0.15, & 0.04 \end{bmatrix}$$

$$\text{cont\_matrix\_before} = \begin{bmatrix} 0.06, & 0.1, & 0.2, & -0.03 \\ 0.12, & -0.05, & 0.15, & 0.04 \end{bmatrix} \otimes \begin{bmatrix} 1 \\ 1 \end{bmatrix} = \begin{bmatrix} 0.06, & 0.1, & 0.2, & -0.03 \\ 0.12, & -0.05, & 0.15, & 0.04 \end{bmatrix}$$

---

ln = 1

$$\text{cont\_matrix\_this} = [0.3,\ 0.5] \bullet \begin{bmatrix} 0.06, & 0.1, & 0.2, & -0.03 \\ 0.12, & -0.05, & 0.15, & 0.04 \end{bmatrix} = [0.078,\ 0.005,\ 0.315,\ 0.011]$$

$$\text{cont\_matrix\_before} = [0.078,\ 0.005,\ 0.315,\ 0.011] \otimes [1] = [0.078,\ 0.005,\ 0.315,\ 0.011]$$

```
cont_list = cont_matrix_before
RETURN(cont_list)
```

```
cont_list = [0.078, 0.005, 0.315, 0.011]

** sum([0.078, 0.005, 0.315, 0.011]) = 0.229
```

Final output y = 0.229

Contribution of x_1, x_2, x_3, and x_4 is [0.078, 0.005, 0.315, 0.011]

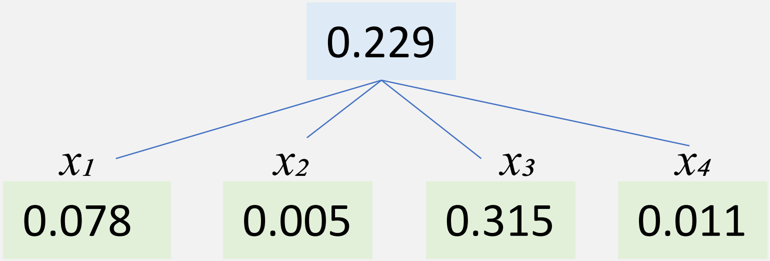

Supplement: Supplemental Information 1 [file peerj-cs-11-2802-s001.pdf]
